# Delaying memory decline: different options and emerging solutions

**DOI:** 10.1038/s41398-020-0697-x

**Published:** 2020-01-21

**Authors:** Felicitas Schneider, Alan Horowitz, Klaus-Peter Lesch, Thomas Dandekar

**Affiliations:** 1 Department of Bioinformatics, Biocenter, University of Würzburg, 97074 Würzburg, Germany; 2 Division of Molecular Psychiatry, Laboratory of Translational Neuroscience, Center of Mental Health, University of Würzburg, Würzburg, Germany; 3 Laboratory of Psychiatric Neurobiology, Institute of Molecular Medicine, I.M. Sechenov First Moscow State Medical University, Moscow, Russia; 4 Department of Psychiatry and Psychology, School for Mental Health and Neuroscience (MHeNS), Maastricht University, Maastricht, The Netherlands; 5 EMBL, Computational Biology and Structures Program, 69117 Heidelberg, Germany

**Keywords:** Long-term memory, Clinical pharmacology, Molecular neuroscience

## Abstract

Memory decline can be a devastating disease and increases in aging Western populations. Memory enhancement technologies hold promise for this and other conditions. Approaches include stem cell transplantation, which improved memory in several animal studies as well as vaccination against Alzheimer´s disease (AD) by β-amyloid antibodies. For a positive clinical effect, the vaccine should probably be administered over a long period of time and before amyloid pathologies manifest in the brain. Different drugs, such as erythropoietin or antiplatelet therapy, improve memory in neuropsychiatric diseases or AD or at least in animal studies. Omega-3 polyunsaturated fatty acid-rich diets improve memory through the gut–brain axis by altering the gut flora through probiotics. Sports, dancing, and memory techniques (e.g., Method of Loci) utilize behavioral approaches for memory enhancement, and were effective in several studies. Augmented reality (AR) is an auspicious way for enhancing memory in real time. Future approaches may include memory prosthesis for head-injured patients and light therapy for restoring memory in AD. Memory enhancement in humans in health and disease holds big promises for the future. Memory training helps only in mild or no impairment. Clinical application requires further investigation.

## Introduction

Memory enhancement is the augmentation of the brain's memory with behavioral, technical or pharmacological methods, for both disease-afflicted and healthy individuals. They target the three main components of memory: encoding, storage, and retrieval, while also bearing on categories such as short/long term, declarative, non-declarative memory, etc. In neurodegenerative disease, there are therapies that treat the symptoms of memory loss, while others attack the fundamental disease. Drugs affecting several physiological systems (e.g., glucocorticoid, endocannabinoid, norepinephrine, dopamine, and serotonin systems) sometimes push different memory functions in opposite directions, for example, enhancing memory formation while impairing retrieval, or vice versa. It is, therefore, critical to determine which functions should be improved and then to select the appropriate approach.

We here focus mainly on methods addressing long term memory processes. Long term memory stores information for a long period of time and can be classified into declarative and procedural memory systems^1^. While procedural memory facilitates learning skills that can be retrieved without conscious awareness, declarative memory enables one to remember personal events (episodic memory) or general information (semantic memory) and frequently requires a conscious recall process^1^. From an anatomical point of view, procedural memory is supported by the striatum and cerebellum^2^. In contrast, declarative memory processes like encoding and consolidation take place in the medial temporal lobe, especially in the hippocampus^3^.

In nature, the different systems of memory cannot be seen as separated from each other but rather interact frequently^4^. Nevertheless, we will describe some examples of how memory-enhancing techniques influence different memory types. Memory loss in early Alzheimer’s disease (AD) mostly affects episodic memory^5^ which represents a part of the declarative long-term memory. Thus, light stimulation, an innovative approach for restoring memory in AD that has been tested in mice, targets episodic memory by activating special hippocampal engram cells^5^. Administration of erythropoietin (EPO) addresses declarative long term memory as well: EPO enhanced long-term potentiation in the hippocampus during picture recall in healthy volunteers^6^ and improved verbal memory mediated through an increase in hippocampal volume in mood disorders^7^. When using mnemonic learning strategies, such as the ancient method of loci (MOL) for remembering items as numbers, faces, and snowflakes, activation of the right hippocampus and other brain regions crucial for spatial memory could be demonstrated^8^. Hence, the MOL seems to address long term memory and spatial memory.

**Fig. 1:**
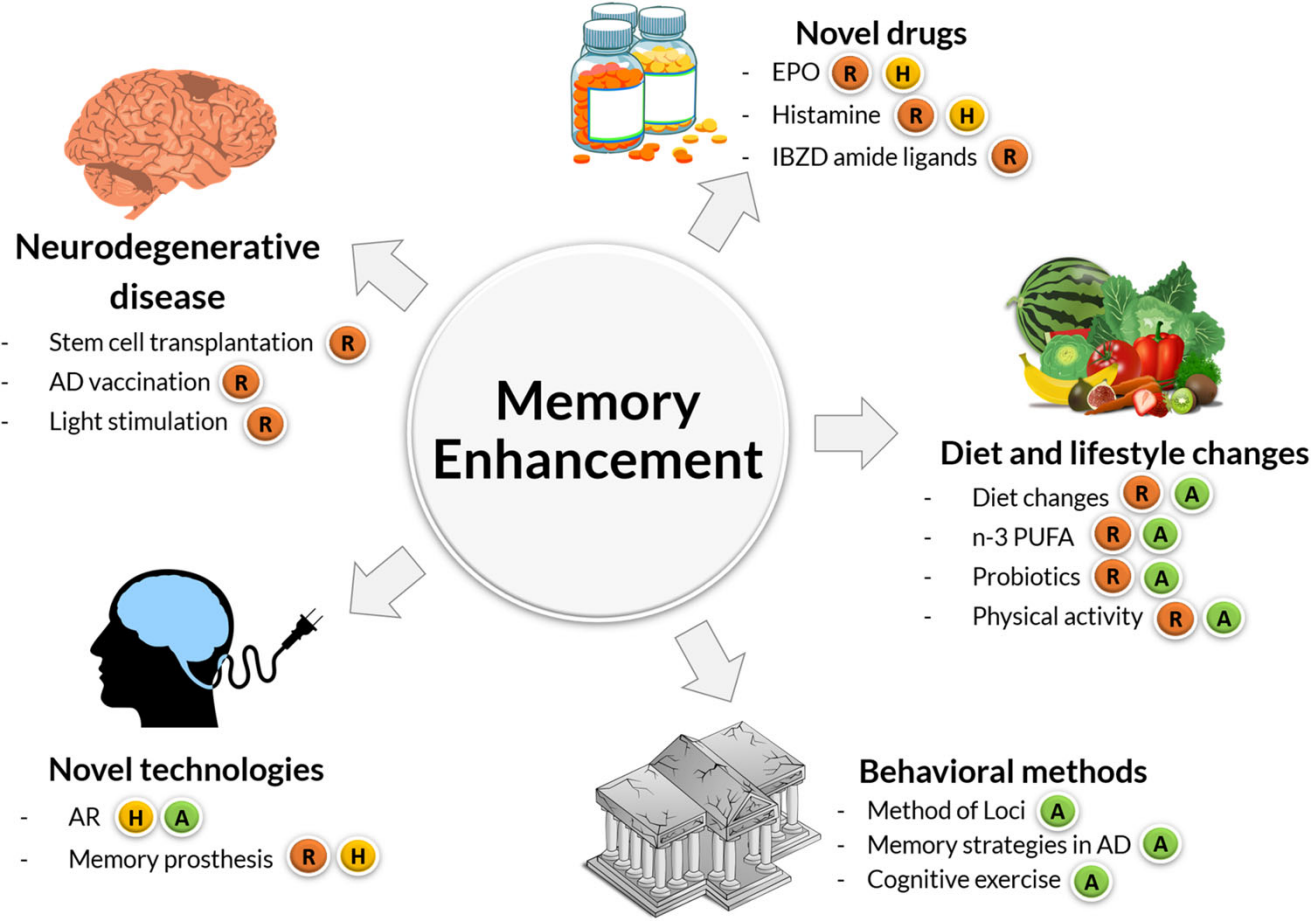
Current approaches for memory enhancement. Five different approaches are explored: novel drugs, diet and lifestyle changes, behavioral methods, novel methods, and direct medical treatment of neurodegenerative disease. Color code indicates R = works in rodents; H = works in humans; A = available treatment.

In this review, we will summarize current approaches for memory enhancement in both diseased and healthy individuals with focus on methods addressing long term memory processes. Our aim is not to be comprehensive, but to critically examine specific prevailing techniques and to discuss some promising concepts for the future (Fig. 1).

Our perspective analyzes first the recent progress in memory-enhancing approaches in AD, such as stem cell transplantation and vaccination against AD. Then, we investigate some rather unconventional neuroenhancement drugs, whose brain function-improving action was just detected in the last few years and might serve both healthy and diseased individuals. These include histamine, EPO, probiotics, and omega-3 polyunsaturated fatty acids (n-3 PUFA). Next, we explore memory techniques and in particular, the ancient “method of loci” (MOL) which mainly stimulates memory capabilities only in the healthy state. To conclude, we investigate augmented reality (AR) and chip devices as a potential booster of learning capacities.

We compare the approaches regarding memory-enhancing effects, briefly describe their physiological background and critically reexamine the prevailing limitations. Regarding the future, we discuss the recovery of memories through light stimulation^5^ and potential memory prostheses^9^.

## Preventing memory decline in neurodegenerative disease

AD is the most common form of dementia with prevalence increasing worldwide^10^. It is characterized by an accumulation of amyloid-ß (Aß) plaques and neurofibrillary tangles (tau protein). The key symptoms consist of cognitive dysfunction in the form of learning impairments as well as memory loss that becomes increasingly severe over time, correlating with extensive neuronal and synaptic losses^11^. So far, the treatment options for reversing the pathologies and neuronal death in AD and other forms like Lewy body^12^ and alcohol-abuse-associated dementia^13^ have been very limited, but in several studies, stem cell therapy showed modestly promising results in rescuing memory.

Stem cells are characterized by their capability to differentiate into different cell types and in the brain were originally thought to replace the damaged neurons in neurodegenerative diseases. Indeed, mesenchymal stem cells derived from human umbilical cord could be shown to transform into nerve-like cells^14^. However, many studies found totally different mechanisms of action including targeting the misfolded proteins or neurogenesis^15^ by, for example, increasing neurotrophins^16^.

One study that used neural stem cells from fetal brain tissue transplanted into two different transgenic mouse models of AD, has shown a significant improvement in cognition, specifically in memory consolidation and to a lesser extent in learning a new task^11^. Interestingly, the stem cells did not alter Aβ or tau protein aggregations, but improved markers that are associated with synaptic integrity and thus, probably synaptogenesis^11^. This effect could have been mediated by BDNF, a neurotrophic factor that is known to enhance synaptic growth^11^.

Another study applied neural stem cell transplantation in a transgenic mouse model of Lewy body dementia (LBD). This treatment was found to correlate with significant improvement in multiple cognitive and motor functions^12^.

Similar to AD, LBD is characterized by the accumulation of a misfolded protein. However, the pathology of α-synuclein, forming Lewy bodies, was not improved by stem cell transplantation. Impaired dopaminergic and glutamatergic signaling after depletion of synaptic storages was restored via a BDNF-dependent mechanism^12^.

Mesenchymal stem cells of bone marrow and human umbilical cord help in neurodegenerative pathologies^17^ including impairment of learning and memory^13,18^ such as in stroke.

A meta-analysis of effects of stem cell transplantation on cognitive function in AD in 58 different studies reinforces a potential benefit regarding learning and memory recovery in experimental settings^10^. Nevertheless, due to several limitations and the poor quality-standards of most of these studies, the estimated positive effect may be overrated^10^. A majority of the better-designed studies found a lower impact on learning and memory^10^. Despite the lesser effect in the better-designed studies, transplantation of bone marrow-derived mesenchymal stem cells did consistently ameliorate Aβ-induced neurotoxicity and cognitive decline in vivo and in vitro. It inhibited apoptotic cell death and oxidative stress in the hippocampus^9^.

Over the last decade, the idea of treating AD by vaccination has also garnered more attention. Immunization against the pathological key players in AD would be an ideal treatment, because the disease process itself could be prevented or at least attenuated^19^. Main strategies of AD vaccination rely on lowering the levels of the pathological Aß peptide^20^ and tau protein^19^. These may contribute to neuronal dysfunction and death^21^ as characteristic features of AD. Indeed, previous studies have shown that both active and passive immune therapies are able to effectively clear β-amyloid deposits in the brains of multiple mouse models of AD^20,22^ and even can improve behavioral deficits^22^. However, these findings did not correlate with a positive clinical outcome in phase three trials with Alzheimer’s patients^20–24^. Nevertheless, a beneficial effect on cognitive functions could be demonstrated in pre-symptomatic or early AD patients treated with passive and active immunization against Aß^20,25^. In the first studies using active anti-Aß immunization, the patients who responded to the therapy achieved significantly better composite scores of memory function and had a slower cognitive decline^20^. In another study using the same vaccine, the Aß burden correlated inversely with the patients’ antibody titer, but there was no clinical effect^20^. There is a long road to a real clinical improvement through an AD vaccination with β-amyloid antibodies. This currently represents the most advanced approach. However, to be effective, the treatment has to be administered before plaque deposition and before the first symptoms of the disease occur^21^. Moreover, the antibody administration must be long-term in order to produce the desired effects^21^.

A promising new vaccination technique for long-term treatment was successfully tested in two mouse models of AD: Cells that continuously secreted β-amyloid antibodies for more than 10 months were implanted subcutaneously before an actual plaque accumulation and dramatically reduced amyloid and tau pathologies^21,25^.

How far a vaccination against AD will be able not only to reverse pathologies, but can actually help to prevent or ameliorate the symptoms of disease clinically, is still not clear^26^. Maybe, despite representing the key findings in AD, the amyloid and tau pathologies do not contribute to the memory and cognitive decline to the extent expected^26^. For instance, the signaling involved in vascular dysfunction in AD was studied and it was found that antiplatelet therapy may, in fact, be helpful as inhibition of platelet signaling alleviates fibril formation in AD patients^27^.

Further studies beyond animal models, especially controlled clinical trials, are required.

Regarding the patho-mechanisms in AD on a cellular level, up to now, many studies suggested that the occurring memory deficits are due to an impaired storage of information^5^. The Tonegawa group recently discovered that an AD mouse model had not forgotten information—their traces were still stored in specific hippocampal cells, so-called engram cells—but were unable to access the information because of disease-related disrupted neuronal signaling^5^. Interestingly, light therapy, mimicking the natural cellular signal, allowed the memories to be retrieved^5^. This optogenetic stimulation of long-term potentiation even succeeded in recovering spine density and long-term memory^5^. In our opinion, these findings contribute tremendously to a deeper insight into the operating principle of human memory itself, as well as the patho-mechanisms of AD. In the last decades, major efforts and large financial expenditures have been made in order to find significant effects of neural stem cell transplantation in neurodegenerative disease, particularly in animal models^28^. Nevertheless, we see only little progress in this field, as compared to other approaches and doubt that the transplantation of stem cells will soon work in humans and become a widely applicable therapy^29^. The handful of therapies currently available provide only transient benefits and the focus should be more on neurotrophins.

In contrast, the relatively new and innovative approach of an Alzheimer’s vaccination has hardly been researched and should be investigated more deeply.

However, the stimulation of memory engram cells via optical long-term potentiation opens a completely new, promising target for future treatments of AD and in our opinion, should be intensely promoted in future research.

## Novel drugs for memory enhancement

Indeed, “classical” neuroenhancement drugs, such as methylphenidate and modafinil actually improve memory^30^, and negative side effects such as abuse are comparatively low.

Similarly, a systematic review tackling newer active drugs for cognitive enhancement found a memory ameliorating effect of melatonin and acetyl salicylic acid, but also admits that most of the studied drugs showed negative results^31^.

We, therefore, would like to report on some novel drugs that are generally not categorized as memory-enhancing. We first focus on EPO as a powerful treatment option in different mental and neurodegenerative diseases that are accompanied by memory deficits.

Better known as a doping drug in sports, increasing physical performance by boosting the number of red blood cells, the hormone EPO has been shown to additionally offer cognition-enhancing and brain-mass-protecting effects independently of its hematopoietic role^7,32,33^. In the last few years, a positive impact on cognition and hippocampal long-term potentiation in healthy human volunteers and rodents was found^6,34^. In a study on EPO as a treatment option for memory impairment in neuropsychiatric diseases, recombinant human EPO could consistently improve memory and cognition in patients with multiple sclerosis, schizophrenia, major depression, and bipolar disorder^7,33,35–37^. While most of the studies on depression even demonstrated a beneficial impact on mood and behavioral symptoms^38,39^, this did not correlate with improvement in memory function^38^, and other investigations did not confirm an antidepressant-like effect^7,40,41^. However, EPO produced a mood-independent enhancement of verbal memory even 6 weeks after treatment completion when red blood cell levels were again normal.

The exact underlying neurobiological mechanisms of EPO are still not completely understood^32^. According to current knowledge, EPO seems to be involved in different modes of action including the promotion of adult neurogenesis^42^ and the release of BDNF^43^.

After EPO treatment, Ehrenreich and colleagues found adult neurogenesis increased by 20% from preexisting neuronal precursors that can differentiate into mature hippocampal memory neurons in a specific hippocampal region^32^. It is important to understand that here EPO acts not as a direct drug for neuroenhancement but rather stimulates differentiation in neuroblasts for adult neurogenesis. This alternative paradigm was based on the results of animal experiments and in vitro experiments using human cell cultures.

Reactivating neuro-regeneration is indeed promising in all cases in which there is an indication/risk of increased and irreversible neuron loss.

Application in healthy subjects in a clinical study is, however, rare. A recent report shows in a case-control study for very preterm infants treated with EPO positive effects in the frontal and temporal lobes and better neuronal connectivity^44^.

Another possible memory-enhancing agent that is better known as a mediator in allergies, is histamine. In a recent study in Japan, the administration of histamine receptor agonists that raised histamine levels and neuronal activity in specific brain regions, improved long term memory in mice^45^. Histamine release enabled the reactivation of neurons that were already firing during memorization^45^. Accordingly, application of histamine agonists could boost memory in healthy human volunteers^45^. These findings open up a completely new field of histamine treatment that, so far, is mostly used to block histamine release via antihistamines. However, to determine whether and in which conditions pro-histamine medications could be applied for memory enhancement, further studies are required.

Lately, a group of researchers in Toronto has developed several new molecules, so-called imidazobenzodiazepine (IBZD) amide ligands that are derived from benzodiazepine (BZ)^46^. IBZD amide ligands target specific gamma-aminobutyric acid (GABA) receptors in brain regions critical for cognition, such as the hippocampus^46^. In a recent study, IBZD amide ligands have been shown for the first time to reverse stress- and age-related working memory impairments in mice^46^. The application of IBZD amide ligands for memory enhancement could be broad, provided that clinical trials confirm the safety and efficacy of these novel molecules. If successfully tested in humans, utilization of IBZD amide ligands for impeding cognitive deficits in AD and mental illnesses such as depression and/or age-related memory loss are conceivable^46^.

## Environment: microbiome, diet, and lifestyle changes

Externally administered drugs have beneficial effects on memory, however, what about internal effectors? Here the gut microbiome and bacterial secreted components, in general, are important to consider. In addition, we will consider diet and lifestyle impacts. The microbiota-gut–brain axis is a recent field of considerable research interest. Comprising trillions of bacteria stimulating afferent neurons of the enteric nervous system^47^, the complex role of the gut microbiome and its effects on the brain are just beginning to be illuminated. There is growing evidence that the composition and diversity of the gut habitat are capable of altering learning, memory, and behavior via different pathways^48–52^.

Even neuropsychiatric diseases like anxiety disorders and depression, as well as age and dementia-related cognitive decline could already be attenuated in animals and man^51,53–55^.

Currently, multiple mechanisms have been proposed on how bacteria in our gut could be able to influence our central nervous system. One theory suggests intestinal permeability due to toxins produced by pathogenic bacteria, as a major route^56^. According to this understanding/theory, such a “leaky gut” could modulate the brain through the release of inflammatory cytokines, or stimulation of the vagal nerve as well as translocation of different metabolic products^56^.

Furthermore, Quintana and his colleagues recently uncovered how gut bacteria products might limit inflammation and neurodegeneration in the central nervous system by influencing microglia and astrocytes^57^. In a mouse model of multiple sclerosis (MS), they demonstrated how the breakdown products of dietary tryptophan produced by microbes directly reduce brain inflammation via the microglial and astrocytic aryl hydrocarbon receptor^57^. In addition, studying human MS brain tissues, the researchers detected similar players and pathways^57^. Besides giving hope for new MS therapies, these findings deliver the first molecular insights on how the gut–brain-connection might work on a mechanistic level.

There are also observations of direct manipulation of the central nervous system by food compounds^58^. However, these effects on the brain could at least in part again be caused by changes in the gut microbiota provoked by nutrition. In one mouse study, a meat-rich diet, known to induce shifts in bacterial variety, was associated with a significant increase in the diversity of the gut flora as well as memory improvement and reduction of anxiety levels^58^. In other animal studies, high fat and particularly high sugar diets were found to deteriorate memory and learning while promoting anxiety by altering the microbiome^50,59,60^. However, a diet rich in polysaturated fatty acids, as opposed to saturated, was not associated with such memory impairments despite a similar caloric intake^60^.

Furthermore, n-3 PUFA mediates some of these beneficial effects on the brain via the gut^61,62^. n-3 PUFA supplementation could be shown to improve performance in different memory tasks in mice, since it provides a variety of neuroprotective effects and induces adult hippocampal neogenesis^62^.

However, inconsistent outcomes in clinical trials of efficacy in neurodegenerative diseases like AD, show that for decisions on concrete dietary measures such as increasing omega-3 polyunsaturated fatty acids more studies are required^63^.

Besides direct dietary modifications, directly altering the gut flora by administering probiotics also shows promise: in animal studies, cognitive impairment due to diabetes mellitus was reversed and learning and memory were improved after probiotic administration^64,65^.

Another factor detrimental for memory retrieval implying bad memory access are mood disorders such as depression or anxiety. In this respect, symptoms of depression, and anxiety were reduced by probiotic supplementation^49,65,66^. The gut microbiome may even be an important factor influencing anxiety, depression and autism and by this lead to memory or mental impairment by ref. ^67^.

A strong and very effective way to improve memory, mnemonic discrimination, flexibility, and spatial working memory is through sports and physical activity^68^. Hence, lifestyles with physical activity and bodily exercise preserve memory and cognition.

In summary, mechanistic insights are available regarding the gut-microbiome-brain axis and there is solid evidence of how lifestyles with physical activity and bodily exercise preserve memory and cognition, and prevent memory decline. Soft and natural interventions such as diet and behavior, including n-3 PUFA, probiotics, and physical activity are effective in preventing and ameliorating symptoms as well as delaying the onset of memory decline. Molecular neuro-regeneration such as through EPO administration, however, requires more basic research before being transferrable to clinical settings. The same applies to translating the influence of the gut microbiome on neuro-inflammation and neuro-regeneration into clinical settings.

## Behavioral methods to enhance memory

There are some behavioral methods that every one of us can easily incorporate into daily life to improve learning and memory that help at the same time in learning impairment by brain injury such as the modified Story Memory Technique^69^. A variety of techniques was already used by the ancient Greeks and Romans that enabled them to accurately deliver long speeches from memory or recall lists of items^60^. The so-called “method of loci“, represents one of the oldest and most effective mnemonic techniques. It consists of first forming mental images of the information to be remembered. Then, one mentally walks along a predefined route through a familiar environment such as one’s own living room, while pictures are stored along the path in the right order^70–72^. For recall, the route is mentally walked in the same manner, thereby recalling the learned items in the correct sequence^70–72^. A study using virtual environments demonstrated that connecting the virtual objects to fixed places along the route seems to be critical for an optimal learning effect^73^. World leading memory athletes, such as Alexander Mullen and Johannes Mallow, use the MOL (A. Mullen remembers 52 playing cards in the right order in only 19.4 s; J. Mallow memorized 504 numbers in no more than 5 min). MOL improved recall for students of an endocrinology class compared to self-directed learning^74^, and expanded memory, brain activation, and even cortical thickness in older adults^75^. Interestingly, MOL was at least as effective as a survival scenario, which is known to boost memory in an outstanding manner^76^. MOL works well also when using unfamiliar, virtual spaces instead of well-known environments^72,77^. Here, they used “memory palaces”, (a technical term for a large, house-like virtual environment to present memory items) implementing the MOL via head-mounted virtual reality devices leading to better recall performances than only displaying them on a traditional desktop^77^. On a mechanistic level, applying mnemonic strategies like the MOL, both by experts and naïve users, leads to transient activation of visuospatial brain regions^8^ and in the long-term, even provokes reorganisation of the functional brain network as a whole^78^.

In superior memorizers using the MOL, none of the brain areas that were activated during memorizing were specifically active during recall^79^. These findings contradict former theories postulating that the brain areas for encoding and retrieval are strongly associated^80^.

London taxi drivers who are not using specific memory techniques to remember London’s 25,000 streets, develop increased posterior hippocampal volume proportional to their navigation expertise^81^. In contrast to drivers using the MOL, they show compromised ability in acquiring new information with a spatial component^81^.

The improvement of memory through spatial strategies might be due to hippocampal networks that help in memorizing a large amount of spatial information in a short period of time^82^.

So-called place and grid cells, that are located in the hippocampal-entorhinal region, store information about the current position and time and may also support declarative memory^82,83^. Spatial strategies obviously facilitate memory. This may be due to spatial codes underpinning human thinking. There is accumulating evidence^79^ that place and grid cells not only encode positions, but encode also dimensions of experience beyond Euclidean space for memory navigation. This implies a more general role of hippocampal-entorhinal processing mechanisms in cognition.

Other memory strategies developed for patients with AD include external memory aids like calendars and diaries, visual imagery, the errorless learning approach, and the spaced retrieval technique^84^. While the efficacy of external memory aids and visual imagery remains questionable, the errorless learning approach and spaced retrieval technique seem to be promising memory training methods for patients with AD^84^.

Apart from memory strategies, there are a variety of other cognitive training techniques aimed at memory improvement. Cognitive exercises, for example, which are clearly distinguished from memory strategies, rely on repeated practice of cognitive tasks instead of incorporating specific learning strategies^85^. Interestingly, a systematic review of subjects with mild cognitive impairment revealed that cognitive exercises might even lead to a greater memory-enhancing effect than memory strategies^85^. This might be due to the activation of multiple brain areas during cognitive exercise, which could be stimulating neuronal plasticity^85^.

In general, there is currently only little knowledge of how such behavioral based mnemonic methods like the MOL exactly succeed in enhancing human memory and if they could also be beneficial against AD and age-related deficits. Moreover, the MOL represents a simple and safe technique to use for anyone in everyday life for more demanding memory tasks. However, adequate adaptation of this and other cognitive training techniques for translation to clinic and patients, for instance, to prevent memory decline in early Alzheimer’s cases, warrants more research and should be a focus of future studies.

## Augmented reality and chip devices to boost memory

AR is a technology that integrates virtual objects and information into the real world and allows the user to interact with these overlaid digital graphics in real time^86^. In contrast, virtual reality (VR) displays complete virtual environments^87^ and is already a great subject for memory research^88^. As memory is environment-dependent, VR offers the possibility to assess and possibly even ameliorate memory impairments in brain damage rehabilitation^89^. Particularly in memory investigations using functional magnetic resonance imaging (fMRI), VR allows the simulation of real-world settings and could thereby reduce distortions of test results^90^. AR is already showing its great potential in the medical education field^91,92^ and hence, seems to be a promising approach for other future learning and education environments, too.

It is not clear yet, how AR exactly might influence our memory, but visualization of the learned material seems to play an important role. A mobile AR tool for memorizing suggests that the main effect is in reducing the cognitive load and hence the exerted learning effort^93^. This finding is supported by a small sample size study, in which the students remembered significantly more medical content than the control group learning with textbooks^94^.

In addition, the usage of instructions and the assembly of objects in the work environment also embodies a practical cognitive-enhancing application domain of AR: In one trial, AR training helped people learn an assembly procedure faster and even better than by utilizing a 3D manual^95^. In another study, the participants assembled a LEGO model quicker than a control group using a paper manual. Moreover, they made fewer mistakes while exerting less effort^96^.

Remarkably, the AR technology may also help against disease-induced cognitive decline like AD. Despite only a very low number of undertaken studies, there is evidence that this new technology could help AD patients by implementing notes and relevant information into their daily routine as virtual reminders in their real living environment^97^.

Using VR or at least AR, serious games (SGs) have some potential to be new and effective tools in the management and treatment of cognitive impairments^98^. In particular, while the patient plays the serious game, these authors developed a battery of tools that are easy to use, reduce the amount of data processing, and provide controllable test conditions for screening, monitoring, and even training pre-dementia condition patients. Another study demonstrated that AR can assist subjects with cognitive impairment in navigation and so possibly contribute to sustaining their independence in everyday life^99^. The physiological mechanisms behind AR are overall similar to gamification strategies: immediate sensory feedback and enhanced memory stimulation from a game-like setting or direct memory and perception task training^100^.

Such combinations are central in understanding how specific memories are formed and improved using AR. AR may hold further beneficial surprises for us and certainly represents a promising opportunity not only for future treatment options in disease and maybe also in enhancing cognitive functions in the daily routines of healthy subjects.

Mixed reality-based (MR) technology combines features of augmented reality with virtual reality. A pilot study tested 21 individuals with mild cognitive impairment (MCI) and MR training (half an hour 3× a week for 6 weeks; *n* = 10) against controls (*n* = 11). The MR training showed significantly improved performance in visuospatial working memory^101^. Similarly, Cogné et al. used visual cueing by directional arrows and salient landmarks^102^.

Spatial navigation and memory tasks were trained in AD (*n* = 20) and MCI patients (*n* = 18) as well as controls (*n* = 20). This showed good results using augmented reality in real-life settings with improved navigational capabilities in the cognitive impaired patients. A recent review sees potential but advocates more controlled studies and larger sample size. Ten studies (5 min to 6 months) compared virtual (*n* = 9) and augmented reality (*n* = 1) in patients with dementia (6 studies) or mild cognitive impairment (3 studies) and 1 study tested both conditions^103^. However, a clear result of the meta-analysis was that virtual experiences were enjoyed by the participants and improved their mood and apathy, and were preferred to other training experiences.

Stimulation, training, and learning by augmented reality depends on sufficient remaining activation potential of memory and brain cells in the diseased patients. In contrast, availability of direct storage like in computers could help directly and always replace fading memory. Hence, a huge possible player in terms of future neuro-enhancement could be the development of a memory prosthesis. Already in 2013, specific firing patterns have been successfully transferred from trained rats into naïve recipient animals via electrical stimulation of hippocampal neurons^104^. Thereby, as shown in the paper, functional working memory of the recipient rats could be improved^104^. Now, two research groups are testing real-time brain stimulation through implanted electrodes in humans^9,105,106^. The first positive results suggest that this cutting-edge development could one day help individuals following brain trauma or head injuries to transfer information from short-term into long-term memory as well as considerably increase retrieval performance^9,105,106^. The key is to derive a nonlinear multi-input, multi-output model of hippocampal CA3 and CA1 neural firing. This allows then accurate electrical stimulation delivered to the same CA1 locations during the sample phase of a delayed match-to-sample task. This then facilitates working memory and subsequently long-term memory^107^.

The research group around Theodore Berger at the University of Southern California in Los Angeles already succeeded in restoring memory-related behavior in rats^108^ and macaques^109^. They bypassed parts of the pathways in the brain critical for forming memories by implanting electrodes that performed the signaling normally done in the brain regions themselves^108,109^.

There are first reports on a real memory chip, so providing data storage for a human brain in neuron readable format for instance by computer-assisted training and augmented reality concepts^77,78^ or genetic readable format^110^. In the latter, information is stored in DNA by light-gated polymerases on a biocompatible nanocellulose support. Read-in and read-out work well in vitro but direct information transfer in neuronal cultures still needs to be shown. However, most convincing is a recent DARPA supported study on a prosthetic memory system stimulating hippocampal CA3 and CA1 neural firing which improved short and long-term memory, as measured by retention of visual information by an average of 35 percent^107^. The brain interface is critical for any of these forms of neuro-enhancement or even memory prosthesis. Startup companies such as Kernel, a neurotech company in Venice (CA) founded by Bryan Johnson collect interested multidisciplinary talents to achieve technological breakthroughs needed for making compact brain interfaces with unprecedented performance a reality for radical human cognitive improvement.

## Discussion

Approaches like stem cell transplantation and vaccination in AD work on a cellular or molecular level in the laboratory^10,21,25^. However, translation into clinical settings will remain a challenge for the next decade. Regarding stem cells, the main problem is the aging patient and tissue including bad vascular support and the short life span of stem cells outside the laboratory. Vaccination in AD has to cope with serious inflammatory side-effects. Improved methods for prevention are required. However, given the molecular achievements so far, there is huge potential for a memory prothesis, the augmented reality approach, and the activation of memory traces by means of light therapy^5^. In our opinion, particularly the idea of transferring the animal findings of Tonegawa to humans in order to enhance memory recall in AD patients by stimulation of specific hippocampal engram cells has great potential and should be pushed intensely forward as a first basis to achieve clinical neuro-enhancement. EPO as a non-classical neuro-enhancement drug, for instance, has been showing impressive memory and cognition results in a variety of neuropsychiatric diseases^7,33,35–37^ and certainly should be further investigated, especially in clinical studies and possibly also with the healthy state in view.

Traditional methods like a microbiome-friendly and n-3 PUFA-rich diet, as well as mnemo-techniques (e.g., MOL) provide good examples that already today effectively enhance cognition but are currently only promising as preventive measures in healthy people^58,61,62,64,65,74^ and potentially in early stages of memory decline. For this too, we strongly advise more research on preventive medicine and treatment of early disease stages. The effects of dietary modifications and nutritional supplements are often hyped in the media, not least because of economic interests. Hence, expecting them to heal memory deficits in severe conditions like AD is unrealistic.
